# Usefulness of the CHAMPS score for risk stratification in lower gastrointestinal bleeding

**DOI:** 10.1038/s41598-022-11666-y

**Published:** 2022-05-09

**Authors:** Munehiko Tajika, Tamotsu Matsuhashi, Yosuke Shimodaira, Sho Fukuda, Tsuyotoshi Tsuji, Kae Sugawara, Youhei Saruta, Yasutaka Takahashi, Kenta Watanabe, Katsunori Iijima

**Affiliations:** 1 Department of Gastroenterology, Akita University Graduate School of Medicine, 1-1-1 Hondo, Akita, 010-8543 Japan; 2 Department of Gastroenterology, Akita City Hospital, Akita, Japan

**Keywords:** Lower gastrointestinal bleeding, Gastrointestinal bleeding

## Abstract

We have recently developed a simple prediction score, the CHAMPS score, to predict in-hospital mortality in patients with upper gastrointestinal bleeding. In this study, the primary outcome of this study was the usefulness of the CHAMPS score for predicting in-hospital mortality with lower gastrointestinal bleeding (LGIB). Consecutive adult patients who were hospitalized with LGIB at two tertiary academic medical centers from 2015 to 2020 were retrospectively enrolled. The performance for predicting outcomes with CHAMPS score was assessed by a receiver operating characteristic curve analysis, and compared with four existing scores. In 387 patients enrolled in this study, 39 (10.1%) of whom died during the hospitalization. The CHAMPS score showed good performance in predicting in-hospital mortality in LGIB patients with an AUC (95% confidence interval) of 0.80 (0.73–0.87), which was significantly higher in comparison to the existing scores. The risk of in-hospital mortality as predicted by the CHAMPS score was shown: low risk (score ≤ 1), 1.8%; intermediate risk (score 2 or 3), 15.8%; and high risk (score ≥ 4), 37.1%. The CHAMPS score is useful for predicting in-hospital mortality in patients with LGIB.

## Introduction

Despite advances in endoscopic and radiologic techniques, gastrointestinal bleeding (GIB) is still associated with significant morbidity and mortality^1,2^. GIB is classified into upper gastrointestinal bleeding (UGIB) and lower gastrointestinal bleeding (LGIB) depending on the anatomic location of the bleeding source. Although there are some differences in the topical treatment (e.g., hemostasis through esophagogastroduodenoscopy vs. colonoscopy, the need for a computed tomography (CT) examination at presentation, or the administration of proton pump inhibitors), other systemic treatments (e.g., blood transfusion, treatment for comorbidities or accompanying complicated diseases, etc.) are largely common, irrespective of the bleeding source. In addition, an accurate diagnosis of the location of bleeding cannot always be made, especially at the initial presentation.

Some risk stratification scores have been developed to predict a variety of clinically relevant outcomes for patients with GIB^3–5^; however, many of these are used to predict the need for hospitalization or the need for endoscopic intervention, and the discriminative performance for predicting the most important outcome, mortality, is relatively poor^6,7^. Furthermore, although these scoring systems could be mainly applied to patients with UGIB, scoring systems for LGIB are relatively scarce^7^. As noted in recent reports, scores that could be applied to both UGIB and LGIB at the same time would be useful for clinicians, since it may be challenging to make an accurate diagnosis of the bleeding location at presentation^8,9^.

Scores that do not require data on endoscopic findings specific to UGIB (pre-endoscopic scores) could be applied to not only UGIB but also LGIB; thus, they can be used to assess the risk of all GIB patients^7^. Indeed, Although the Glasgow-Blatchford score (GBS), clinical Rockall score (cRS), and AIMS65 scores, all of which are pre-endoscopic scores^3–5^, were initially designed for UGIB, some reports have indicated that these scores are also useful for the risk stratification of LGIB^10–13^. Recently, a new pre-endoscopic score, the ABC score was developed for predicting mortality in patients with overall GIB early after presentation, and showed high accuracy for both UGIB and LGIB^9^.

We have recently developed a simple prediction score, the CHAMPS score using the clinical data of 2205 subjects with UGIB, to predict in-hospital mortality based on six easily available clinical variables^14,15^. The score successfully discriminated patients with UGIB who were at risk for in-hospital mortality with an AUC of 0.90 for derivation cohort, and 0.81 for the validation cohort, and had had significantly higher discriminative ability than the GBS, cRS, AIMS65 score, and ABC score^14^. In addition, the CHAMPS score can be applied to patients with UGIB, irrespective of the status of hospitalization (outpatient onset and inpatient onset)^14^, although most of the previous scores could only be applied to the outpatient status at the development of UGIB^14^. Furthermore, since the CHAMPS score does not require endoscopic findings^14^, the score could be applied to not only patients with UGIB but also patients with LGIB.

In this study, we retrospectively investigated whether the CHAMPS score, which was originally designed for risk stratification of UGIB, could also be useful for LGIB in comparison to the other above-mentioned scores.

## Results

**Figure 1:**
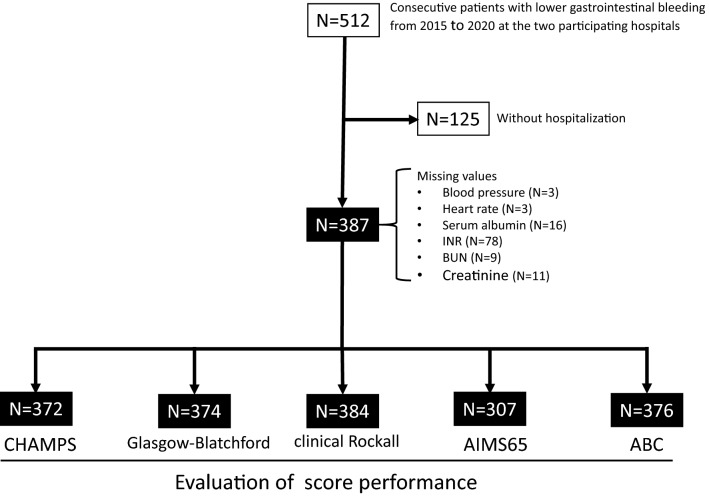
Flow chart of patient selection in each scoring system. The performance of each scoring system for predicting outcomes was evaluated in all cases with necessary data, but comparisons of performance among scoring systems were only evaluated in matched cases.

Initially, 512 patients with LGIB were identified in the two participating hospitals during the study period. Among these patients, 125 who did not require hospitalization due to mild bleeding were excluded, leaving 387 patients with LGIB as study subjects for this analysis (Fig. 1). The characteristic features of the 387 patients and comparisons between survivors and non-survivors are shown in Table 1. Based on colonoscopy and CT examination, the diagnoses of patients with LGIB were as follows: presumptive or definitive diverticular bleeding, n = 132 (34.1%); rectal ulcer, n = 72 (18.6%); ischemic colitis, n = 54 (13.9%); delayed post-polypectomy–induced bleeding, n = 23 (5.9%), hemorrhoid bleeding, n = 21 (5.4%); bleeding colonic cancer, n = 18 (4.7%); bleeding colitis, n = 13 (3.4%); telangiectasia, n = 8 (2.1%), and unknown/other, n = 32 (8.2%). Stigmata of recent hemorrhage was observed in 140 (36.2%) patients.

**Table 1:** Characteristics of enrolled subjects with lower gastrointestinal bleeding.

	Total (n = 387)	Survival (n = 348)	Death (n = 39)	*P* value
Sex: male	223 (57.6%)	195 (56.0%)	28 (71.8%)	0.054
Age: years	75 (64–83)	71.5 (63–82)	75.8 (64–87)	0.078
In-hospital onset	106 (27.4%)	80 (23.0%)	26 (66.7%)	< .00001
ECOG-PS	1 (0–2)	1 (0–2)	2 (1–4)	< .0001
Charlson Comorbidity Index	2 (0–3)	2 (0–3)	3 (2–5)	0.0001
Altered mental status	20 (5.2%)	18 (5.2%)	2 (5.1%)	1.0
Systolic blood pressure: mmHg (n = 384)†	125 (106–142)	125.7 (108–144)	109 (96.7–122)	< 0.0001
Heart rate: /min. (n = 384)†	81 (70–95)	82.6 (70–95)	86.4 (72–99)	0.20
**Blood test**				
Hemoglobin: g/dL	11 (8.7–13.2)	11.0 (8.9–13.3)	9.4 (7.3–11)	0.001
Serum Albumin: g/dL (n = 371)†	3.4 (2.9–4.0)	3.5 (3–4)	2.4 (1.9–3)	< .00001
INR (n = 308)†	1.06 (0.96–1.3)	1.3 (0.9–1.2)	1.3 (1.1–1.5)	0.84
BUN: mg/dl (n = 378)†	19.0 (13.8–28.7)	23.3 (13.7–28.2)	31.9 (15.7–41.9)	0.0044
Creatinine: mg/dl (n = 376)†	0.8 (0.6–1.1)	1.1 (0.6–1.1)	1.5 (0.7–1.7)	0.074
**Medication**				
Non-steroidal anti-inflammatory drugs: yes	60 (15.5%)	50 (14.7%)	10 (25.6%)	0.098
Anti-thrombotics: yes	82 (21.2%)	71(20.4%)	11 (28.2%)	0.30
Steroid: yes	36 (9.3%)	30 (8.6%)	6 (15.4%)	0.24
Stigma of recent hemorrhage: yes	140 (36.2%)	123 (35.3%)	17 (43.6%)	0.38
Re-bleeding: yes	33 (8.5%)	30 (8.6%)	3 (7.7%)	1.0
Emergency CT: yes	104 (26.9%)	99 (28.4%)	5 (12.8%)	0.037
**Scoring system**				
CHAMPS score (n = 372)†	1 (0–2)	1 (0–2)	3 (2–4)	< .00001
Glasgow-Blatchford score (n = 374)†	5.5 (2–9)	1 (1–2)	2 (1–3)	0.0001
Clinical Rockall score (n = 384)†	3 (2–4)	3 (1–4)	4 (2–5)	< .00001
AIMS65 score (n = 307)†	1 (1–2)	5 (2–9)	8 (5–11)	0.0011
ABC score (n = 376)†	2 (1–5)	2 (1–5)	4 (2–7)	0.0052

Continuous variables were expressed in the median and interquartile range, and categorical valuables were expressed as the number and proportion. †: there are some cases with missing values. ECOG-PS: Eastern Cooperative Oncology Group Performance Status; INR: International normalized ratio; BUN: Blood urea nitrogen.

**Figure 2:**
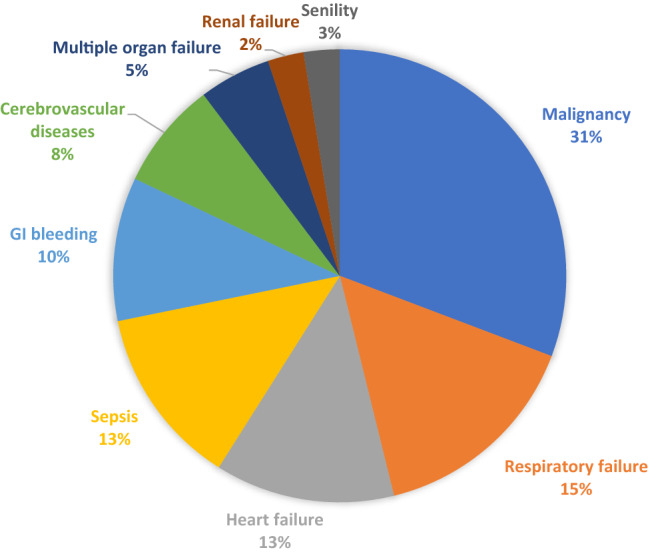
Causes of death in 39 cases with a fatal outcome.

Overall, 39 (10.1%) patients died during the index hospitalization. Among these, only 4 (10.2%) died directly from uncontrolled bleeding, and the majority (89.8%) died from non-bleeding causes. The details of the cause of death are shown in Fig. 2. Meanwhile, 33 (8.5%) experienced re-bleeding during the index hospitalization.

**Figure 3:**
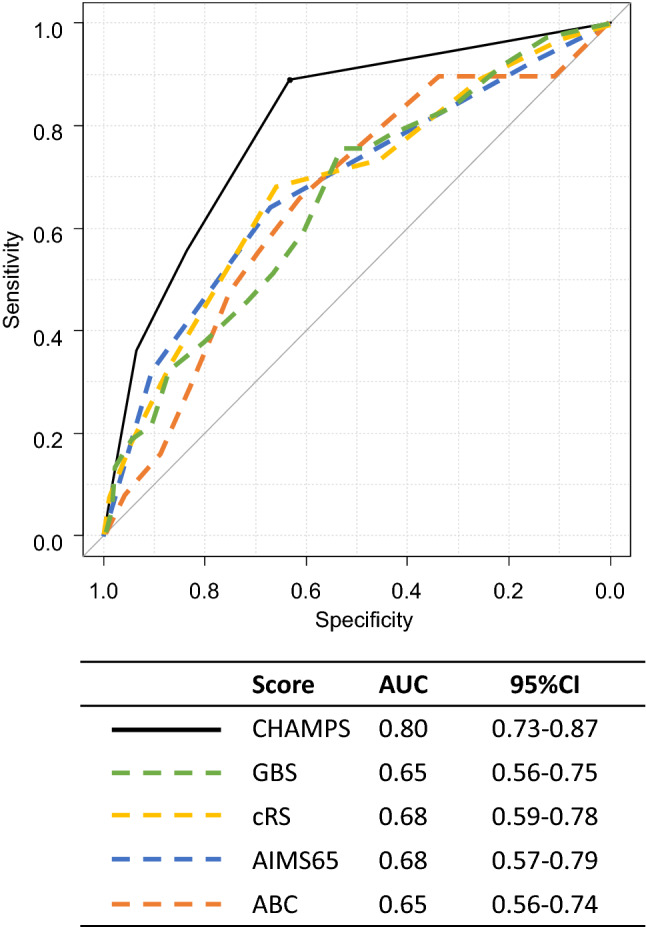
Receiver operating characteristic curves comparing the performance of the five scoring systems in the prediction of in-hospital mortality. AUC, area under the receiver operating characteristic curve; CI, confidence interval; cRS, clinical Rockall score; GBS, Glasgow-Blatchford score.

The median CHAMPS score, GBS, cRS, AIMS65 score, and ABC score for each eligible patient was 1 (0–2), 5.5 (2–9), 3 (2–4), 1 (1–2), and 2 (1–5), respectively. ROCs comparing the performance of these 5 scoring systems in predicting in-hospital mortality are shown in Fig. 3. The CHAMPS score showed good performance in the prediction of in-hospital mortality in LGIB patients with an AUC (95% CI) of 0.80 (0.73–0.87). The performance of the CHAMPS score was significantly superior to the GBS (AUC 0.66, 95% CI 0.56–0.75, *P* < 0.01), cRS (AUC 0.68, 95%CI 0.59–0.78, *P* < 0.01), and ABC score (AUC 0.65, 95%CI 0.56–0.74, *P* < 0.001), and was marginally superior to that of the AIMS65 score (AUC 0.68, 95% CI 0.57–0.79, *P* = 0.08). The addition of the status of the presence or absence of rebleeding (point 1 or 0) to the CHAMPS score (CHAMPS-R score)^14^ made it numerically inferior to the original CHAMPS score with regard to its ability to predict in-hospital mortality (AUC 0.78, 95% CI 0.71–0.86) (data not shown). The CHAMPS score showed a high AUC in patients with either definite or presumptive source of LGIB with an AUC (95% CI) of 0.76 (0.67–0.86) or 0.84 (0.73–0.95), respectively. In addition, AUCs with 5 scores are shown in Supplemental Table 1 separately in patients with outpatient onset or inpatient onset.

The rate of in-hospital mortality in patients with a CHAMPS score of 0, 1, 2, 3, 4, and ≥ 5 were 1.8%, 1.9%, 15.0%, 17.5%, 37.0%, and 37.5%, respectively. Thus, in-hospital mortality increased in a three-stepwise manner, and was categorized into 3 risk groups as follows: low risk (score 0 or 1), 1.8%; intermediate risk (score 2 or 3), 15.8%; and high risk (score ≥ 4), 37.1% (Table 2).

**Table 2:** Distribution of risk scores and risk classification in CHAMPS score for in-hospital mortality in patients with lower gastrointestinal bleeding.

Risk score (6-point scoring system)				Risk classification			
Total points	Patients (*n* = 372)†	In-hospital mortality (*n* = 36)	Rate of in-hospital mortality, % (95% CI)	Risk category	Patients (*n* = 372)†	In-hospital mortality (*n* = 36)	Rate of in-hospital mortality, % (95% CI)
0	109	2	1.8 (0.2–6.5)	Low	217	4	1.8 (0.2–6.5)
1	108	2	1.9 (0.2–6.5)				
2	80	12	15.0 (8.0–24.7)	Intermediate	120	19	15.8 (10.3–23.5)
3	40	7	17.5 (7.3–32.8)				
4	27	10	37 (19.4–57.6)	High	35	13	37.1 (23.1–53.7)
5	8	3	37.5 (8.5–75.5)				
6	0	0	0				

^†^There were missing data in 15 cases. CI, confidence interval.

The sensitivity, specificity, PPV, NPV, and accuracy of the CHAMPS and the other 4 scores in predicting a low or high risk of in-hospital mortality are shown in Table 3. The CHAMPS score categorized a relatively high proportion (58.3%) of patients as low risk, and showed good specificity and excellent PPV with an overall accuracy of 55.7% for predicting low-risk patients. Further, the CHAMPS score classified a small portion (9.4%) of patients as being at high risk, and it showed excellent specificity and NPV with overall accuracy of 87.9% for predicting high-risk patients. In the remaining (32.3%) intermediate-risk patients, the CHAMPS score showed 52.8% sensitivity and 69.9% specificity with an overall accuracy of 68.3%. Thus, among the 5 investigated scoring systems, the CHAMPS score showed the highest overall accuracy in predicting high-risk patients.

**Table 3:** Comparison of diagnostic ability for in-hospital mortality among five prediction scores in patients with lower gastrointestinal bleeding.

	Prediction scores				
	CHAMPS score	GBS	cRS	AIMS65 score	ABC score
AUC (95% CI)	0.80 (0.73.–0.87)	0.66 (0.56–0.75)	0.68 (0.59–0.78)	0.68 (0.57–0.79)	0.65 (0.56–0.74)
**Low-risk†**					
Patients, *n* (%)	217 (58.3)	74 (19.8)	33 (8.6)	197 (64.2)	251 (66.8)
Mortality, *n* (%)	4 (1.8)	3 (4.1)	1 (3.0)	10 (16.4)	17 (6.8)
Sensitivity, %	11.1	8.1	2.6	35.7	44.7
Specificity, %	58.8	78.9	90.8	33.0	30.8
PPV, %	1.8	4.1	3.0	5.1	6.8
NPV, %	90.5	88.7	89.5	83.6	83.2
Accuracy, %	55.7	71.9	82.0	33.2	32.2
**High-risk‡**					
Patients, *n* (%)	35 (9.4)	204 (54.5)	217 (56.5)	110 (35.8)	45 (12.0)
Mortality, *n* (%)	13 (37.1)	28 (13.7)	28 (12.9)	18 (16.4)	6 (13.3)
Sensitivity, %	36.1	75.7	73.7	64.3	15.8
Specificity, %	93.5	47.8	45.4	67.0	88.5
PPV, %	37.1	13.7	12.9	16.4	13.3
NPV, %	93.2	94.7	94.0	94.9	90.3
Accuracy, %	87.9	50.5	48.2	66.8	81.1

*AUC* area under the receiver operating characteristic curve, *GBS* Glasgow Blatchford score, *cRS* clinical Rockall score, *CI* confidence interval, *PPV* positive predictive value, *NPV* negative predictive value.

^†^Low-risk, CHAMPS scores = 0, 1; GBS ≤ 1; cRS = 0; AIMS65 score ≤ 1; and ABC score ≤ 3.

^‡^High-risk, CHAMPS scores ≥ 4; GBS ≥ 5; cRS ≥ 3; AIMS65 score ≥ 2; and ABC score ≥ 8.

The ROCs to compare the performance of the CHAMPS score, GBS, cRS, AIMS65 score, and ABC score in predicting rebleeding are shown in Supplemental Fig. 1. The CHAMPS score showed only modest performance in predicting rebleeding in LGIB patients with an AUC (95% CI) of 0.67 (0.57–0.77); the other 4 scoring systems showed similarly modest performance with AUCs ranging from 0.57 to 0.67 (Supplemental Fig. 1).

## Discussion

We recently developed a simple, pre-endoscopic score (CHAMPS score) with high discriminative ability for predicting in-hospital mortality in patients with UGIB^14^. Furthermore, the current study demonstrated that the CHAMPS score also has high discriminative ability (AUC: 0.80) for predicting in-hospital mortality in patients with LGIB, with a significantly higher AUC in comparison to the other existing scores. Thus, the CHAMPS score should be useful for the earlier prediction of the most important outcome, death, in all patients with GIB.

In this study, the rate of all-cause in-hospital mortality in patients with LGIB was 10.1%, which was substantially higher than that in previous studies (3–4%)^2,16,17^. The exclusion of mild LGIB, which was not required for hospitalization, should be partly responsible for the higher in-hospital mortality observed in this study. Alternatively, this study included cases of both outpatient- and inpatient-onset LGIB, while many previous studies only included outpatient-onset LGIB. This could be another reason for the high mortality in this study since LGIB with an onset during hospitalization is known to be associated with higher mortality in comparison to outpatient-onset LGIB^2,18,19^. In any case, the present study was consistent with previous studies dealing with UGIB or LGIB patients^1,2,15,20^, in that it demonstrated that uncontrolled bleeding accounted for the cause of death in a small portion (10%) of the cases in which patients died after LGIB, with bleeding-unrelated causes accounting for the vast majority (90%) of the remaining deaths. This finding reinforces the importance of general intensive management for patients with GIB to improve their overall prognosis, irrespective of the source of bleeding (UGIB or LGIB), without merely focusing on the local control of bleeding.

Fifty-eight percent of LGIB patients were categorized as low-risk by the CHAMPS score. These patients showed a low rate (1.8%) of in-hospital mortality which was lower in comparison to the other scoring systems that were investigated (3.0–16.4%). For these patients, although they required hospitalization for LGIB, early discharge could be possible. On the other hand, 9.4% were categorized as high-risk. These patients showed a very high rate (37.1%) of in-hospital mortality, which was much higher than that of the other 4 scores (12.9–16.4%). Since approximately 90% of this group died from the non-bleeding related causes, systematic, intensive care in a specialized unit from the initial stage would be appropriate. Meanwhile, although the remaining 32% of patients were categorized as intermediate-risk, the in-hospital mortality rate was substantial (15.8%); hence, early discharge should be avoided and careful observation is required for these patients. Thus, the CHAMPS score should be useful for the management of LGIB patients depending on their degree of risk at presentation.

GIB can be a life-threatening condition and requires emergent testing and treatment, irrespective of the source of bleeding. Although some different treatment approaches could be applied depending on the source of bleeding (UGIB or LGIB), it is not always easy to distinguish between UGIB and LGIB in patients with hematochezia, especially at presentation. Hence, a scoring system to predict significant outcomes irrespective of the source of bleeding would be useful in considering the initial response of patients with GIB^8,9^. In the present study, we demonstrated that the CHAMPS score is useful for predicting the most important outcome, mortality, in a broad range of GIB patients, irrespective of the bleeding source (UGIB or LGIB) or hospitalization status (outpatient onset or inpatient onset).

In our original study to develop the CHAMPS score for predicting in-hospital mortality in patients with UGIB, the threshold for low-risk patients was set at 0, while that for high-risk patients was set at ≥ 3^14^. Nonetheless, in the current study, which analyzed patients with LGIB, the risk of in-hospital mortality increased in a three-stepwise manner as the CHMPS score increased, and the patients could be categorized into 3 groups accordingly. Then, we modified the thresholds for predicting in-hospital mortality in patients with LGIB, changing the threshold for low-risk to ≤ 1 and that for high-risk to ≥ 4. Actually, the overall accuracy in predicting low-risk and high-risk patients with the modified thresholds of the CHAMPS score seems to be superior to that of the original thresholds (Supplemental Table 2). Although the CHAMPS score itself is useful for predicting in-hospital mortality, irrespective of the source of GIB (UGIB or LGIB), further studies are warranted to investigate whether different thresholds are required for risk stratification between UGIB and LGIB.

The ABC score has recently been developed to predict mortality in both UGIB and LGIB among European populations, and showed good performance in UGIB (AUC: 0.86 and 0.81) and LGIB (AUC: 0.84)^9^. Nonetheless, the ABC score showed rather low performance in the Japanese population in our recent study dealing with UGIB (AUC = 0.77)^14^ and in the current study dealing with LGIB (AUC = 0.65). The discordant performance of the ABC may be partly explained by the ethnic difference (European population vs. Japanese population), as patient age is a significant risk factor for mortality in the former but not in the latter^9,14^. Otherwise, the potential difference in the underlying diseases of LGIB between the two populations may be responsible for the discordant performance. In particular, acute hemorrhagic rectal ulcers, which are much more common in Asian countries and which are associated with a poor prognosis^21,22^, accounted for a substantial portion (19%) of the enrolled LGIB in the current study.

Recently, a risk scoring system (NOBLADS score) was developed to predict severe outcomes (e.g., requirement of blood transfusion, longer hospital stay, and intervention) in Japanese subjects with LGIB^23^. Nonetheless, the additional analysis revealed that the CHAMPS score showed significantly higher discriminatory ability in the prediction of in-hospital mortality in comparison to the NOBLADS score (AUC [95%CI]: CHAMPS, 0.80 [0.73–0.87]; NOBLADS, 0.73 [0.65–0.81]; *P* = 0.02, Supplemental Fig. 2). In addition, although the CHAMPS score was already validated for UGIB, the NOBLADS is unsuitable for predicting the outcomes of UGIB since some of the factors evaluated in the system should apply specifically to patients with LGIB (e.g., diarrhea and abdominal tenderness)^23^. Thus, the CHAMPS score is superior to the NOBLADS score as a single scoring system to predict in-hospital mortality for UGIB and LGIB together.

One limitation of this study may be the relatively low number of enrolled patients. Although clinical data on consecutive patients with LGIB who were managed at two tertiary academic medical centers over a 6-year period were collected with a substantial number of cases with the main outcome (in-hospital mortality), the number of subjects may have been insufficient to draw robust conclusions. Furthermore, several variables, especially INR, had missing values, and we had to exclude those cases from the analysis. Finally, in the current study dealing with LGIB, there were no significant differences in altered mental status or steroid use between survivors and non-survivors, although these two factors were incorporated into the CHAMPS score based on our recent study dealing with UGIB^14^. In this study, we would prioritize applying the same scoring system (CHAMPS score) to both UGIB and LGIB, rather than establishing a different, new score for LIBG alone. Nonetheless, these two factors may not necessarily be required to predict the mortality in LGIB, and indeed, the AUC remained high, even after excluding the two factors from the scoring system (AUC [95%CI]: 0.81 [0.73–0.88]) (data not shown). An additional study is required to further validate the usefulness of the CHAMPS score in the risk stratification of patients with LGIB.

## Conclusion

This study successfully demonstrated that the CHAMPS score, a score originally developed for UGIB, is also useful for predicting in-hospital mortality in patients with LGIB. Thus, the CHAMPS may be useful for risk stratification at presentation in all patients with signs of suspected GIB (e.g., hematemesis, hematochezia, and anemia) irrespective of the source of bleeding.

## Methods

### Patients

Consecutive adult patients who were hospitalized with LGIB from 2015 to 2020 at two tertiary academic medical centers in Akita prefecture in Japan (Akita University Hospital and Akita City Hospital) were retrospectively enrolled in this study. Those with mild LGIB, who did not require hospitalization, were excluded since the main outcome of this study was in-hospital mortality. Patients with hospitalization for LGIB (outpatient onset) or the development of LGIB after hospitalization for another indication (inpatient onset) were both included in the analysis. The indications for hospitalization were clinically significant GIB (e.g., hypotension, shock, orthostatic changes in systolic blood pressure and/or pulse, repeated bleeding, or a > 2 g decrease in hemoglobin from baseline.

### Data collection

The following clinical data, which are required to calculate the CHAMPS score, GBS, cRS, AIMS65 score, and ABC score, were collected^3–6,9,14^: patient demographics (age and sex), in-hospital/out-of-hospital onset, altered mental status, vital signs (systolic blood pressure and pulse), physical condition (Eastern Cooperative Oncology Group Performance Status), comorbid conditions (Charlson Comorbidity Index), blood test (hemoglobin, albumin, and creatinine, international normalized ratio [INR], and blood urea nitrogen [BUN]), and medication (anticoagulants, antiplatelet agents, nonsteroidal anti-inflammatory drugs, and steroids) at the onset of LGIB.

### Data availability

The datasets used and analyzed during the current study available from the corresponding author on reasonable request.

### CHAMPS score

The CHAMPS score is a simple equal-weight score, determined based on six variables (Charlson Comorbidity Index ≥ 2, in-hospital onset, albumin < 2.5 g/dL, altered mental status, Eastern Cooperative Oncology Group performance status ≥ 2, steroid use); the maximum score is six points (Table 4)^14^.

**Table 4:** CHAMPS score comprising equal-weight six variables.

Variables	Points
Charlson Comorbidity Index (≥ 2)	1
In-hospital onset (Yes)	1
Albumin (< 2.5 g/dL)	1
Altered mental status (Yes)	1
Eastern Cooperative Oncology Group Performance Status (≥ 2)	1
Steroids (Yes)	1
Total	6

### Definitions

LGIB was defined as presentation with hematochezia, including red blood or clots per rectum, maroon-colored stool or blood mixed in with stool, but any patient with suspected UGIB at esophagogastroduodenoscopy was excluded. Colonoscopy was performed emergently or electively with or without CT in all patients with LGIB to identify the source of bleeding. Both definite and presumptive sources of LGIB were included. Definite sources of bleeding were defined as lesions with documented visualization of active bleeding, a visible vessel, or adherent clot (stigmata of recent hemorrhage)^24^. Presumptive diagnoses were defined as cases of diverticula, hemorrhoids, or angiodysplasia, without stigmata of recent bleeding^24^.

### Outcomes

The primary outcome of this study was the usefulness of the CHAMPS score for predicting in-hospital mortality in patients with LGIB. This was determined by comparing the CHAMPS score with existing scoring systems. In-hospital mortality was defined as death during the index hospitalization, whatever the cause. The secondary outcome was the efficacy of the CHAMPS score for predicting re-bleeding during the index hospitalization. Re-bleeding was suspected based on the presence of fresh hematochezia and circulating instability after successful hemostasis and was defined as a new bleeding episode from the same source based on an endoscopic examination.

### Ethical considerations

The protocol for this study was approved by the ethics committee of Akita University School of the Medicine (approval number: 2676). All the methods were performed in accordance with relevant guidelines and regulations. The relevant informed consent was obtained from patients enrolled and the study design was published, and patients had a chance to opt out of the use of their information for this study.

### Statistical analyses

Continuous variables were expressed as the median and interquartile range and were compared using the Mann–Whitney U-test. Categorical valuables were expressed as the number and proportion and proportion and were compared using the chi-squared test. The performance of the scoring systems for predicting outcomes was assessed by a receiver operating characteristic curve (ROC) analysis. The area under the receiver operating characteristic curve (AUC) was calculated, and the CHAMPS score was compared with those of four existing scores (GBS, cRS, AIMS65 score, and ABC Score) using the DeLong test. Then, the sensitivity, specificity, positive predictive value (PPV), negative predictive value (NPV), and accuracy of each score were compared. According to the previous studies^3–6,9,25^, the thresholds for low-risk patients were set at ≤ 1, 0, ≤ 1, and ≤ 3 in the GBS, cRS, AIMS65 score, and ABC score, respectively, while those for high-risk patients were set at ≥ 5, ≥ 3, ≥ 2, and ≥ 8. All analyses were conducted using the EZR software program (Saitama Medical Center, Jichi Medical University, Saitama, Japan)^26^, and *P* values of < 0.05 were considered to indicate statistical significance.

## Supplementary Information

Supplementary Information 1.

Supplementary Information 2.

Supplementary Information 3.

## Abbreviations

AUC: Area under the receiver operating characteristic curve
CI: Confidence interval
cRS: Clinical Rockall score
CT: Computer tomography
GBS: Glasgow-Blatchford score
GIB: Gastrointestinal bleeding
LGIB: Lower gastrointestinal bleeding
NPV: Negative predictive value
PPV: Positive predictive value
ROC: Receiver operating characteristic curve
UGIB: Upper gastrointestinal bleeding
