# Supplementary material for: Usefulness of the CHAMPS score for risk stratification in lower gastrointestinal bleeding
Source: Sci Rep. 2022 May 9;12:7587. doi: 10.1038/s41598-022-11666-y (PMC9085815; doi:10.1038/s41598-022-11666-y)
Supplement: Supplementary file 1 — Supplementary Information 1. [file 41598_2022_11666_MOESM1_ESM.docx]

**Supplemental table 1: Diagnostic performance of five scoring systems in the prediction of in-hospital mortality depending on the onset situation (outpatient/inpatient onset)**

| Score | Outpatient onset | |  | Inpatient onset | |
| --- | --- | --- | --- | --- | --- |
|  | AUC | 95% CI |  | AUC | 95% CI |
| CHAMPS | 0.73 | 0.57-0.88 |  | 0.66 | 0.55-0.78 |
| Glasgow-Blatchford | 0.69 | 0.52-0.85 |  | 0.45 | 0.32-0.59 |
| Clinical Rockall | 0.62 | 0.44-0.81 |  | 0.63 | 0.51-0.75 |
| AIMS65 | 0.66 | 0.43-0.89 |  | 0.55 | 0.42-0.69 |
| ABC | 0.62 | 0.42-0.82 |  | 0.56 | 0.45-0.67 |

AUC, area under the receiver operating characteristic curve; CI, confidence interval

**Supplemental table 2: Comparison of diagnostic ability of CHAMPS score using two different thresholds**

|  | CHAMPS score | |
| --- | --- | --- |
|  | Threshold 1 | Threshold 2 |
| Low-risk |  |  |
| Patients, *n* (%) | 217 (58.3) | 109 (29.3) |
| Mortality, *n* (%) | 4 (1.8) | 2 (1.8) |
| Sensitivity, % | 11.1 | 5.6 |
| Specificity, % | 58.8 | 59.3 |
| PPV, % | 1.8 | 1.8 |
| NPV, % | 90.5 | 82.1 |
| Accuracy, % | 55.7 | 52.8 |
|  |  |  |
| High-risk |  |  |
| Patients, *n* (%) | 35 (9.4) | 75 (20.7) |
| Mortality, *n* (%) | 13 (37.1) | 20 (27.4) |
| Sensitivity, % | 36.1 | 55.6 |
| Specificity, % | 93.5 | 83.6 |
| PPV, % | 37.1 | 26.7 |
| NPV, % | 93.2 | 94.6 |
| Accuracy, % | 87.9 | 80.9 |

Threshold 1 (modified): Low-risk, CHAMPS scores =0, 1; High-risk, CHAMPS scores ≥4

Threshold 2 (original): Low-risk, CHAMPS scores =0; High-risk, CHAMPS scores ≥3

PPV, positive predictive value; NPV, negative predictive value
