# Supplementary material for: Usefulness of the CHAMPS score for risk stratification in lower gastrointestinal bleeding
Source: Sci Rep. 2022 May 9;12:7587. doi: 10.1038/s41598-022-11666-y (PMC9085815; doi:10.1038/s41598-022-11666-y)
Supplement: Supplementary file 2 — Supplementary Information 2. [file 41598_2022_11666_MOESM2_ESM.pptx]

## Slide 1
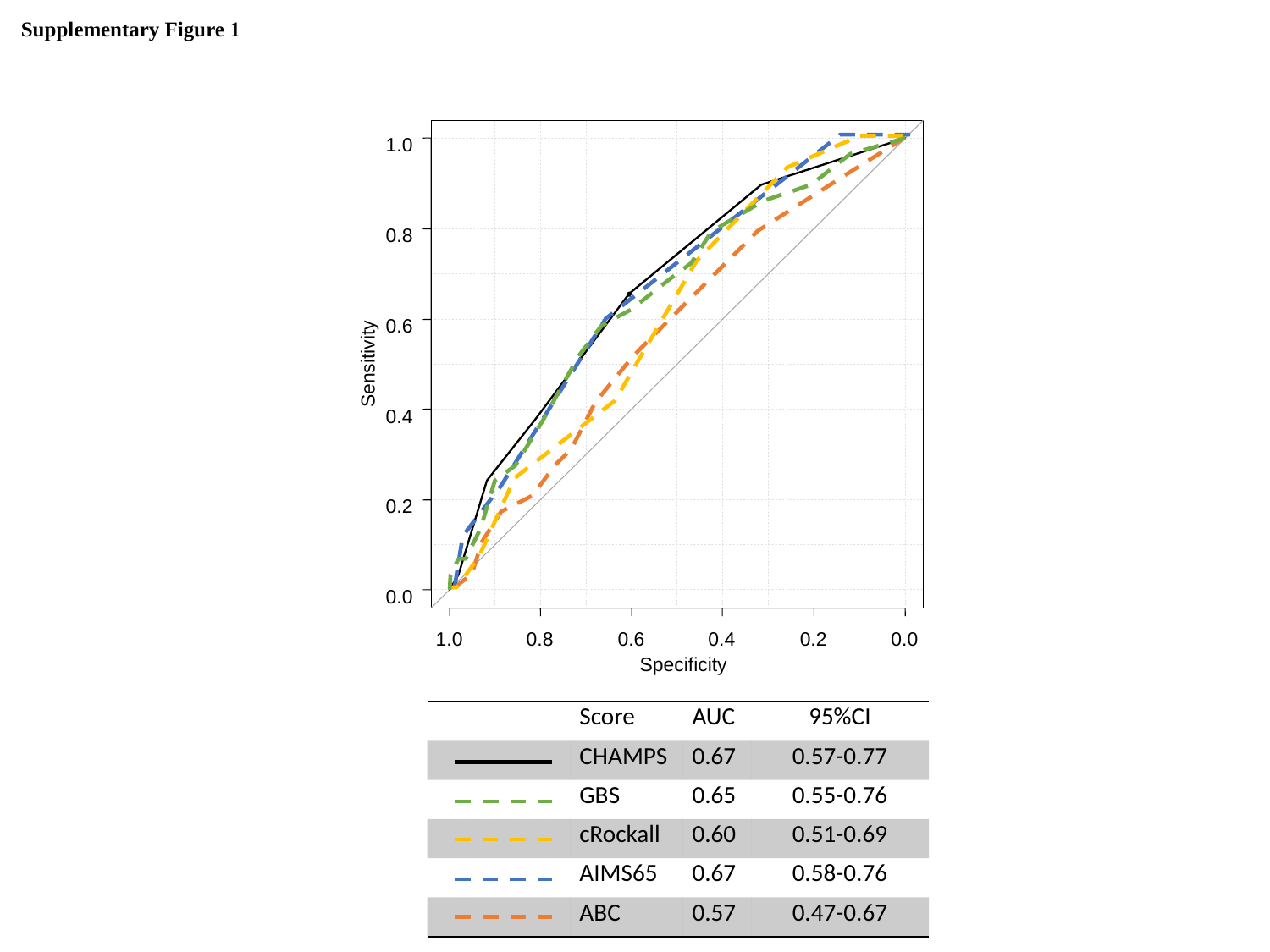

Supplementary Figure 1
1.0
0.8
0.6
Sensitivity
0.4
0.2
0.0
1.0
0.8
0.6
0.4
0.2
0.0
Specificity
| | Score | AUC | 95%CI |
| --- | --- | --- | --- |
| | CHAMPS | 0.67 | 0.57-0.77 |
| | GBS | 0.65 | 0.55-0.76 |
| | cRockall | 0.60 | 0.51-0.69 |
| | AIMS65 | 0.67 | 0.58-0.76 |
| | ABC | 0.57 | 0.47-0.67 |

## Slide 2
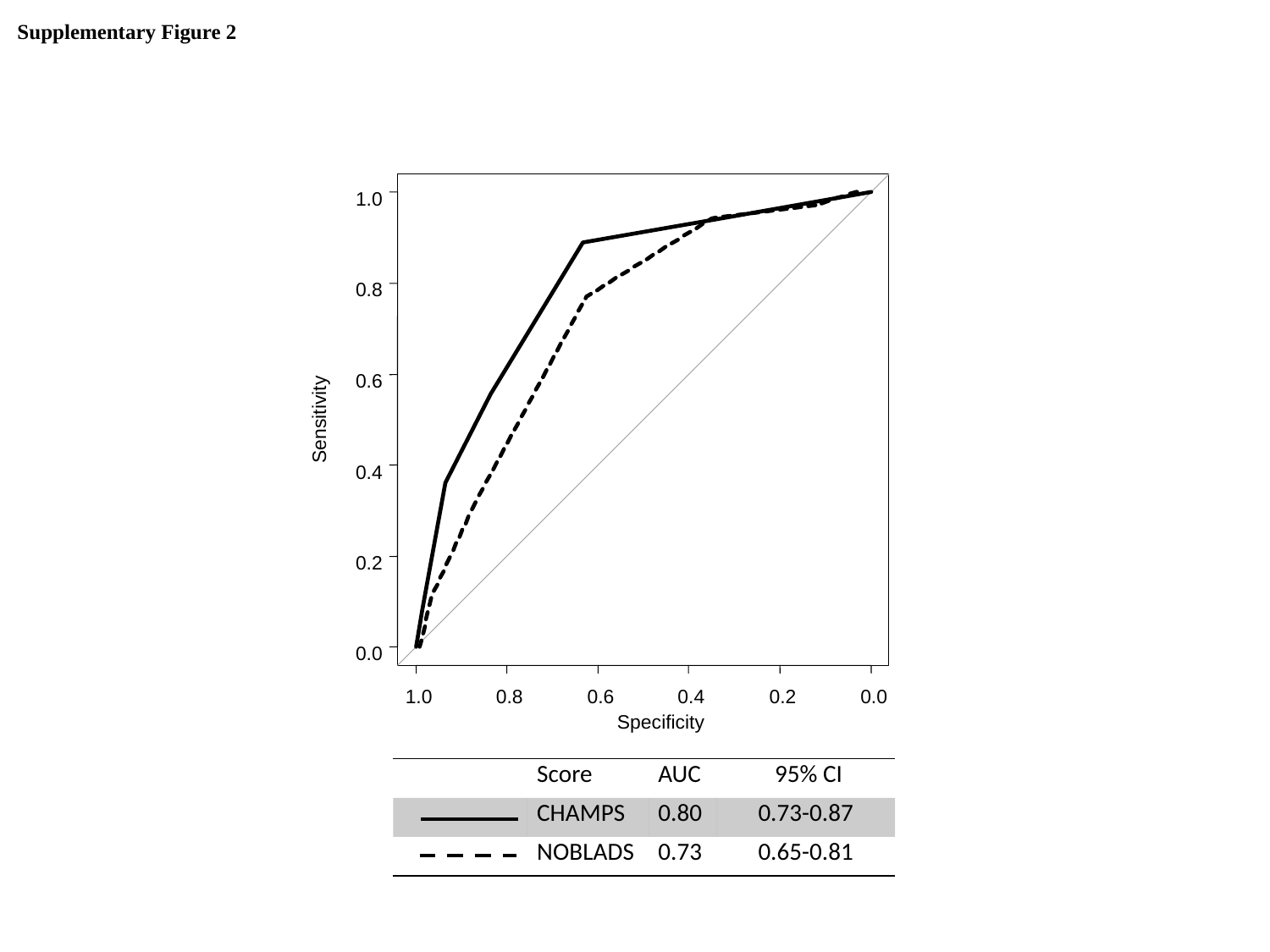

Supplementary Figure 2
1.0
0.8
0.6
Sensitivity
0.4
0.2
0.0
1.0
0.8
0.6
0.4
0.2
0.0
Specificity
| | Score | AUC | 95% CI |
| --- | --- | --- | --- |
| | CHAMPS | 0.80 | 0.73-0.87 |
| | NOBLADS | 0.73 | 0.65-0.81 |
