# Supplementary material for: Usefulness of the CHAMPS score for risk stratification in lower gastrointestinal bleeding
Source: Sci Rep. 2022 May 9;12:7587. doi: 10.1038/s41598-022-11666-y (PMC9085815; doi:10.1038/s41598-022-11666-y)
Supplement: Supplementary file 3 — Supplementary Information 3. [file 41598_2022_11666_MOESM3_ESM.docx]

**SUPPLEMENTARY FIGURE LEGENDS**

**Supplementary Figure 1: Receiver operating characteristic curves comparing the performance of the five scoring systems in the prediction of rebleeding**

AUC, area under the receiver operating characteristic curve; CI, confidence interval; cRS, clinical Rockall score; GBS, Glasgow-Blatchford score. **Supplementary Figure 2: Receiver operating characteristic curves comparing the performance of the CHAMPS score and NPBLADS score in the prediction of rebleeding**

AUC, area under the receiver operating characteristic curve; CI, confidence interval.
